# Treatment with Non-Steroidal Anti-Inflammatory Drugs (NSAIDs) Does Not Affect Outcome in Patients with Acute Myocarditis or Myopericarditis

**DOI:** 10.3390/jcdd9020032

**Published:** 2022-01-19

**Authors:** Moritz Mirna, Lukas Schmutzler, Albert Topf, Elke Boxhammer, Brigitte Sipos, Uta C. Hoppe, Michael Lichtenauer

**Affiliations:** Department of Internal Medicine II, Division of Cardiology, Paracelsus Medical University of Salzburg, 5020 Salzburg, Austria; l.schmutzler5@gmail.com (L.S.); a.topf@salk.at (A.T.); e.boxhammer@salk.at (E.B.); b.sipos@salk.at (B.S.); u.hoppe@salk.at (U.C.H.); m.lichtenauer@salk.at (M.L.)

**Keywords:** cardiology, myocarditis, inflammation, ibuprofen, aspirin, acetylsalicylic acid, NSAID, NSAR

## Abstract

*Background:* Previous animal studies reported an association of non-steroidal anti-inflammatory drugs (NSAIDs) with adverse outcomes in acute myocarditis, which is why these drugs are currently not recommended in affected patients. In this retrospective case-control study, we sought to investigate the effects of NSAID treatment in patients with acute myocarditis and myopericarditis to complement the available evidence. *Method:* A total of 114 patients with acute myocarditis were retrospectively enrolled. Demographical, clinical and laboratory data were extracted from hospital records. Patients who received NSAIDs (*n* = 39, 34.2%) were compared to controls. Follow-up on all-cause mortality was acquired for two years. Propensity score matching was additionally conducted to account for covariate imbalances between groups. *Results:* Treatment with NSAIDs was neither associated with a worse outcome (*p* = 0.115) nor with significant differences in left ventricular systolic function (*p* = 0.228) or in-hospital complications (*p* = 0.507). *Conclusion:* Treatment with NSAIDs was not associated with adverse outcomes in our study cohort. Together with the findings of previous studies, our results indicate that these drugs could be safely administered in patients with myocarditis and myopericarditis.

## 1. Introduction

Non-steroidal anti-inflammatory drugs (NSAIDs) such as acetylsalicylic acid and ibuprofen constitute a cornerstone in the treatment of acute pericarditis, where they have proven effective in ameliorating symptoms and reducing inflammation [1,2,3,4]. In contrast to acute pericarditis, treatment with NSAIDs is currently not recommended in patients with acute myocarditis [5] because of the findings of previous animal studies that suggested deleterious effects of these drugs on disease progression [5,6]. For example, in 1985, Costanzo-Nordin et al. reported that intraperitoneal administration of ibuprofen would aggravate myocardial inflammation and necrosis in BALB/c mice with coxsackievirus B3-induced myocarditis [7]. Similar findings were reported by Khatib et al., who found that treatment with indomethacin increases titers of coxsackisevirus B4 in a mouse model of acute myocarditis [8], and by Rezkalla et al., who reported increased mortality, elevated viral titers and reduced concentrations of interferon in animals treated with NSAIDs [9].

However, since acute pericarditis and acute myocarditis share very similar etiologies, with cardiotropic viruses being the most common cause of both disease entities in the Western world [10], the two overlap syndromes of ‘myopericarditis’ and ‘perimyocarditis’ are frequently encountered in clinical practice [11,12]. Intriguingly, current guidelines by the European Society of Cardiology endorse treatment with NSAIDs in the case of ‘myopericarditis’, despite their reservation in patients with acute myocarditis only [13]. Moreover, the current guideline document states that the application of available evidence from animal studies to humans concerning the use of NSAIDs in acute myocarditis may be questionable, especially in light of the lack of evidence in humans [13]. To the best of our knowledge, two studies have thus far investigated clinical outcomes of patients with myopericarditis treated with NSAIDs [14,15], while only one retrospective case-control study has investigated outcomes of patients treated with NSAIDs in comparison to patients treated with standard heart failure treatment. Interestingly, the authors of the latter study reported that treatment with NSAIDs was associated with a statistically non-significant reduction in late gadolinium enhancement (LGE) in follow-up magnetic resonance imaging (MRI) after three months [16], suggesting a potential benevolent effect of NSAID treatment in these patients.

In light of the lack of evidence concerning the use of NSAIDs in patients with acute myocarditis and myopericarditis, we thus sought to investigate the effects of these drugs on affected patients, especially regarding their impact on long-term mortality, in-hospital complications and left ventricular (LV) systolic function, in order to complement the available evidence and thus facilitate future treatment of our patients.

## 2. Materials and Methods

This study was conducted in accordance with the Declaration of Helsinki and the principles of Good Clinical Practice. The study outline was reviewed and approved by the ethics committee of the state of Salzburg, Austria (EK Nr: 1181/2020), prior to patient enrollment.

### 2.1. Data Collection

We performed a retrospective case-control study of patients admitted to the University Hospital of Salzburg, Austria, in the time period of 2009 to 2019. Eligible patients with myocarditis were identified through discharge diagnoses recorded in hospital discharge forms, which were classified according to the International Classification of Diseases, Tenth Revision (ICD-10) diagnostic codes (I40.0, I40.1, I40.8, I40.9, I51.4). Presence of myocarditis was confirmed by revision of all clinical records, laboratory data, results from cardiac magnetic resonance imaging (MRI) and endomyocardial biopsy (EMB). Patients were only enrolled in the study if they fulfilled the current diagnostic criteria for clinically suspected myocarditis by the European Society of Cardiology (ESC) [5] and had proof of myocardial inflammation on cardiac MRI and/or EMB. Patients admitted for elective procedures and follow-up visits, as well as those with chronic or recurrent myocarditis, were excluded from the study. Patients were included in the non-steroidal anti-inflammatory (NSAID) group if they received a fixed prescription for NSAID treatment due to myocarditis. Patients with on-demand treatment or NSAID use before admittance were also excluded from the study.

Demographical, clinical and laboratory data were extracted from the initial hospital record after admittance, i.e., from the emergency department, the intermediate care unit (IMC) or intensive care unit (ICU), or the hospital ward. Follow-up data on left ventricular ejection fraction (LVEF) were acquired from transthoracic echocardiography (TTE) exams or MRI conducted 90 to 180 days after hospitalization.

### 2.2. Primary and Secondary Study Endpoints

The primary study endpoint was all-cause mortality within the follow-up period of 24 months after presentation. Secondary study endpoints were in-hospital complications (combined endpoint of hemodynamically relevant arrhythmias, respiratory compromise necessitating IMC/ICU admission, cardiogenic shock or in-hospital mortality), as well as fold change of systolic LVEF 90 to 180 days post-hospitalization.

### 2.3. Statistical Analyses

Statistical analyses were performed with SPSS (Version 23.0, IBM, Armonk, NY, USA) and R (version 4.0.2., R Core Team (2013), R Foundation for Statistical Computing, Vienna, Austria; http://www.R-project.org/ accessed on 17 December 2021) using the packages ‘Rcmdr’, ‘ggplot2′, ‘pastecs’, ‘Hmisc’, ‘ggm’, ‘polycor’, ‘QuantPsyc’, ‘glmnet’, ‘Matching’, ‘MatchIt’, ‘optmatch’, ‘RItools’, ‘Rcpp’, ‘stddidff’, ‘jtools’, ‘survival’ and ‘survminer’. Skew, kurtosis and data distribution of continuous data were assessed visually and by applying the Shapiro–Wilk test, whereas homogeneity of variances was assessed by the Levene test. Since most data were not normally distributed, they were depicted as median ± interquartile range (IQR), and medians were compared using the Mann–Whitney U test. Wilcoxon signed-rank test was used to compare medians of paired samples. Categorical data were analyzed by applying Fisher’s exact test. In order to account for covariate imbalances with a possible influence on the outcome, standardized differences between the two groups were additionally calculated. Covariates with statistically significant differences in frequencies or medians of baseline data, or standardized differences of >0.25 between the groups, were then included in the propensity score matching of groups using the ‘nearest neighbor matching’ approach, with a 1:1 ratio and a caliper of 0.25. Prior to matching, numeric data were converted to z-scores to assure standardization of the included covariates. A *p*-value of <0.05 was considered statistically significant.

## 3. Results

### 3.1. Baseline Characteristics

In total, 114 patients with acute myocarditis were enrolled in this study. Of these, 34.2% (*n* = 39) received NSAIDs for a mean duration of 2.9 ± 2.2 weeks, either due to myopericarditis (61.5%, *n* = 24) or acute myocarditis (38.5%, *n* = 15). A total of 75 patients (65.8%) constituted the control group who did not receive NSAIDs.

Baseline characteristics of enrolled patients are depicted in Table 1. Patients who received NSAIDs were significantly younger (median 29 years (IQR 21–38) vs. median 37 years (IQR 27–49), *p* = 0.005) and had higher serum concentrations of C-reactive protein (CRP; median 7.35 mg/dL (IQR 1.45–12.55) vs. median 1.90 mg/dL (IQR 0.60–6.45), *p* = 0.004) at baseline. Furthermore, the peripheral leukocyte count was significantly elevated in these patients (median 9.85 G/L (IQR 7.62–13.11) vs. median 7.87 (IQR 6.49–11.23), *p* = 0.048) when compared to controls. There were no statistically significant differences in relevant comorbidities or LV systolic function (NSAID: median 56% (IQR 50–60) vs. median 55% (IQR 50–60), *p* = 0.378) between the two investigated groups (see Table 1).

Treatment administered for myocarditis/myopericarditis is depicted in Table 2. The most frequently administered NSAID was acetylsalicylic acid (51.3%, *n* = 20), with a mean dose of 1260 ± 395 mg per day. Ibuprofen was administered in 46.2% (*n* = 18) of the patients, while diclofenac was administered in only one patient (2.6%). While colchicine was administered more often in patients receiving NSAIDs (20.5% vs. 1.3%, *p* = 0.001), there were no statistically significant differences in the frequency of steroids, beta blockers, angiotensin-converting enzyme inhibitors (ACEI)/angiotensin receptor blockers (ARB) or mineralocorticoid receptor antagonists (MCRA) between the two investigated groups (see Table 2).

### 3.2. Primary and Secondary Study Endpoints before Propensity Score Matching

#### 3.2.1. All-Cause Mortality within Follow-Up

**Figure 1:**
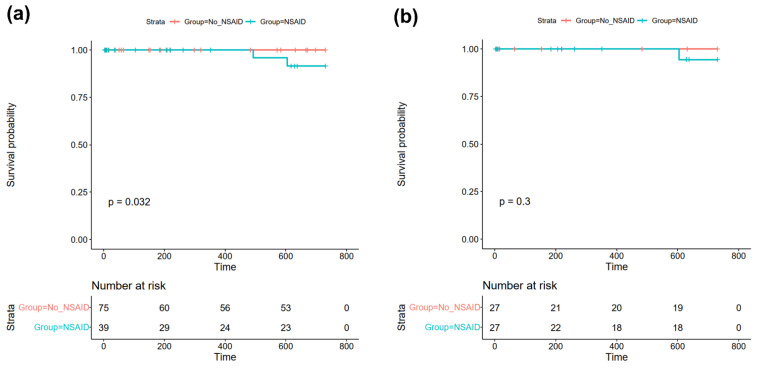
Kaplan–Meier plots of all-cause mortality within 24 months of follow-up in (**a**) the total study cohort and (**b**) the matched cohort after propensity score matching. Abbreviations: NSAID = non-steroidal anti-inflammatory drug.

The mean follow-up was 531 ± 289 days, and complete follow-up for 24 months was available for 69 patients (60.5%). Of the total cohort, two patients died within 24 months of follow-up (1.7% of total cohort), both of whom had received NSAIDs for myopericarditis during their index hospitalization (NSAID: 5.1% vs. No NSAID: 0.0%, *p* = 0.115, see Table 3; log-rank: *p* = 0.032, see Figure 1a and Figure S3). Of these, a female of 42 years died on day 492 of the follow-up because of cardiac decompensation, whereas a male of 73 years died on day 604 because of acute respiratory distress of an unknown cause. In univariate Cox proportional hazard analysis, treatment with NSAIDs was not associated with an increased risk of all-cause mortality (HR 224.0 (95%CI 0.0–225736909.8), *p* = 0.443).

In the subgroup of patients with acute myocarditis only (*n* = 71, 62.3% of the total study cohort), no event of death occurred during follow-up, neither in patients receiving NSAIDs (*n* = 15, 21.1% of subgroup), nor in those who did not receive NSAIDs (*n* = 56, 78.9% of the subgroup).

#### 3.2.2. In-Hospital Complications

Patients who received NSAIDs had a trend towards more in-hospital complications (combined endpoint of hemodynamically relevant arrhythmias, respiratory compromise necessitating IMC/ICU admission, cardiogenic shock or in-hospital mortality); however, this finding remained statistically insignificant (12.8% vs. 8.0%, *p* = 0.507, see Table 3).

The same trend was observed in the subgroup of patients with acute myocarditis only (*n* = 71); however, it also remained statistically insignificant in these patients (NSAID: 13.3% vs. No NSAID: 7.1%, *p* = 0.600).

#### 3.2.3. LVEF after 90–180 Days

**Figure 2:**
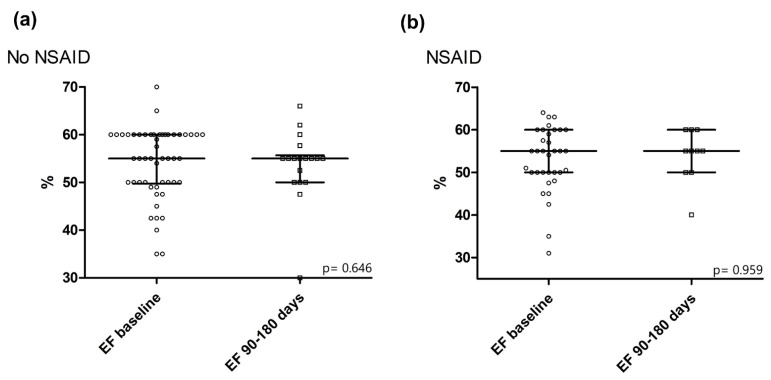
Left ventricular systolic function at baseline and at follow-up 90 to 180 days after hospitalization in (**a**) patients who did not receive NSAIDs and (**b**) patients treated with NSAIDs. Depicted is the *p*-value for the Wilcoxon signed-rank test for paired samples. Abbreviations: EF = ejection fraction, NSAID = non-steroidal anti-inflammatory drug.

**Figure 3:**
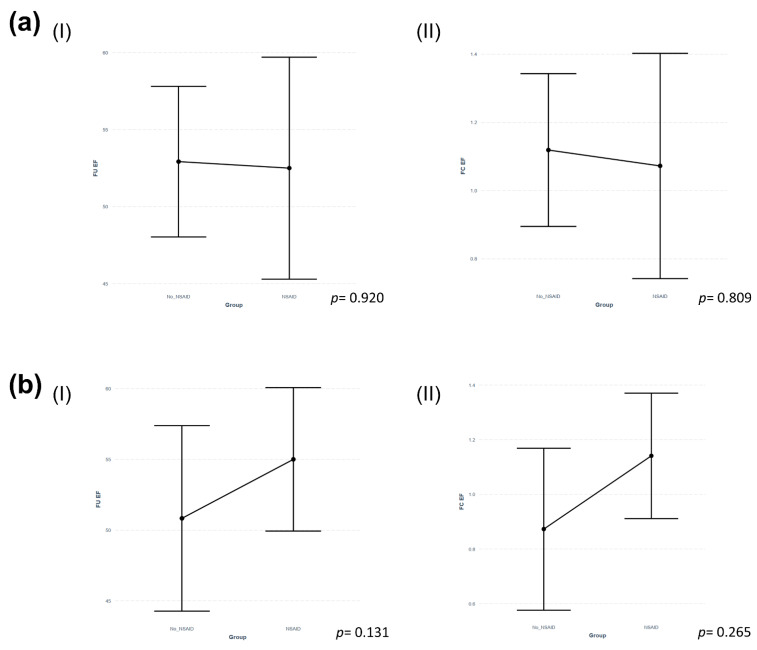
Predictor effect plots of predicted values of univariate linear regression analysis in (**a**) the total study cohort and (**b**) the matched cohort after propensity score matching for (**I**) follow-up LVEF after 90–180 days and (**II**) fold change of EF after 90–180 days. Abbreviations: FC EF = fold change of ejection fraction, FU EF = follow-up ejection fraction, NSAID = non-steroidal anti-inflammatory drug.

There was no statistically significant difference in LV systolic function after 90–180 days between patients of both groups (NSAID: median 55% (IQR 54–60) vs. No NSAID: median 55% (IQR 50–56), *p* = 0.228, see Figure 2). Furthermore, there was no difference in the fold change (FC) of EF from baseline to follow-up after 90–180 days (NSAID: median 1.00 (IQR 0.88–1.14) vs. No NSAID: median 1.03 (IQR 0.92–1.21), *p* = 0.898, see Table 3). In univariate linear regression analysis, treatment with NSAIDs was furthermore neither associated with EF at follow-up (B (SE) = −0.4231 (4.1275), R^2^ = −0.0249, *p* = 0.920), nor with its FC (B (SE) = −0.0465 (0.1891), R^2^ = −0.0595, *p* = 0.809, see Figure 3a, (I) and (II)).

Notably, there was also no statistically significant difference in LV systolic function at follow-up between patients with and without NSAID treatment (NSAID: median 55% vs. No NSAID: median 55%, *p* = 0.280) within the subgroup of patients with acute myocarditis only (*n* = 71).

### 3.3. Primary and Secondary Study Endpoints after Propensity Score Matching

Propensity score matching was conducted to account for covariate imbalances between the two groups that could have had an effect on the outcome. Covariates were included if there were statistically significant differences in frequencies or medians and/or standardized differences of >0.25 between the two groups (arterial hypertension, treatment with beta blockers, treatment with colchicine, age, CRP, leukocyte count; see Figures S1 and S2).

Then, statistical analyses for the primary and secondary study endpoints were repeated in a matched cohort of 27 patients receiving NSAIDs and 27 patients of the control group. One patient treated with NSAIDs from the matched cohort died during follow-up; however, there were no statistically significant differences between both groups regarding all-cause mortality (3.7% vs. 0.0%, *p*-value = 0.500; Cox proportional hazard analysis: HR 69.0 (95%CI 0.0–670,199,590.4, *p* = 0.606; log-rank: *p* = 0.30 see Figure 1b).

Furthermore, there was still a trend towards more in-hospital complications in patients treated with NSAIDs; however, this finding also remained statistically insignificant (NSAID: 11.1% vs. No NSAID: 3.7%, *p* = 0.610). In the matched cohort, treatment with NSAIDs was also neither associated with reduced EF at follow-up (B (SE) = 0.2680 (0.1531), R^2^ = 0.5815, *p* = 0.131), nor with a reduced FC of EF (B (SE) = 4.1677 (3.388), R^2^ = 0.4487, *p* = 0.265, see Figure 3b, (I) and (II)).

## 4. Discussion

With a class IA recommendation in the current ESC guidelines [13], NSAIDs represent the mainstay of therapy in patients with acute pericarditis and should be administered in full doses to all affected patients until symptom resolution [17]. However, despite similar causal pathogens and the frequently encountered overlap syndrome of ‘myopericarditis’, these drugs are currently not recommended in patients with acute myocarditis due to the findings of previous animal studies that suggested negative effects of NSAIDs on disease progression [5,6].

During follow-up, two patients from our study cohort died, resulting in a 24-month mortality of 1.7%. Intriguingly, both of these patients had received NSAIDs for myopericarditis during their index hospitalization, which resulted in a statistically significant difference between Kaplan–Meier curves with *p* = 0.032 in log-rank test (see Figure 1a), despite a non-significant Fisher exact test (5.1% vs. 0.0%, *p* = 0.115) and no association of NSAIDs with mortality in Cox proportional hazard analysis (*p* = 0.443). Since both patients died well after one year of follow-up, and the mean duration of NSAID treatment was only 2.9 ± 2.2 weeks in our study, an association of NSAID use with mortality was considered unlikely. To further investigate the discrepancy between log-rank test and Fisher’s exact test, we chose to conduct propensity score matching in order to account for covariate imbalances with a possible impact on the outcome between the two investigated groups. Here, we did not observe an association with all-cause mortality in the matched cohort, indicating that treatment with NSAIDs does not affect all-cause mortality in patients with acute myopericarditis. This finding is in line with a previous study by Berg et al., where treatment with NSAIDs was also not associated with increased mortality during a mean follow-up of 12.1 ± 9.6 months in patients with myopericarditis [16]. Additionally, no event of death was reported in previous studies by Buiatti et al. [14] and Imazio et al. [15], where the majority of enrolled patients with myopericarditis had received NSAID therapy. Since the three aforementioned studies only investigated patients with myopericarditis, we further performed subgroup analysis of patients with acute myocarditis only (*n* = 71, 62.3% of the total study cohort). No event of death was registered in this subgroup, indicating that treatment with NSAIDs is unlikely to increase mortality in these patients. However, the low number of patients analyzed has to be considered in this regard.

Concerning secondary study endpoints, we did not observe a negative effect of NSAID use on LV systolic function, as indicated by the lack of a statistically significant difference in the medians of EF (see Figure 2a,b) or FC of EF after 90 to 180 days, and the lack of an association of NSAIDs with these parameters in univariate linear regression analysis (also see Figure 3b, (I)).

Together with the findings of previous studies [14,15,16], our results thus indicate that treatment with NSAIDs is not associated with a worse outcome or decreased LV systolic function at follow-up in patients with acute myocarditis or myopericarditis. Conversely, since a previous study reported a non-significant reduction in LGE in follow-up cardiac MRI associated with NSAID treatment [16], these drugs could, in turn, even elicit benevolent effects in affected patients. As such, two recent meta-analyses identified LGE as a strong independent predictor of adverse outcomes in patients with myocarditis, which probably derives from more extensive myocardial damage and fibrosis, with an associated higher risk for ventricular arrhythmias [18,19]. In light of the absence of causal therapeutic approaches [20,21], as well as the current reservation of treatment with anti-inflammatory drugs and corticosteroids in the current ESC guidelines [5], this finding could be of special prognostic implication for patients with myocarditis. Therefore, a large prospective randomized controlled trial is further warranted to elucidate if treatment with NSAIDs has an effect on the outcome in these patients. Here, the statistically non-significant trend towards more in-hospital complications in patients receiving NSAIDs observed in our cohort (12.8% vs. 8.0%, *p* = 0.507, see Table 3) should also be investigated further.

Nevertheless, the potential side effects of treatment with NSAIDs have to be considered when these drugs are administered at full dosages [22,23]. As such, NSAIDs have been associated with gastrointestinal mucosal injury [23], arterial hypertension [24], impairment of renal function [25] and increased cardiovascular morbidity and mortality [26], although they are generally considered safe and effective [27]. Therefore, concise evaluation of the indication and all comorbidities is warranted in patients to whom these drugs should be administered.

With this study, we complement the limited evidence on NSAID therapy in patients with acute myocarditis and myopericarditis, and furthermore, we provide the first propensity-matched analysis in this regard. Interestingly, treatment with NSAIDs was neither associated with a worse outcome nor with a statistically significant difference in LV systolic function or in-hospital complications when compared to controls. Thus, these drugs could provide symptomatic and anti-inflammatory effects in affected patients, which should be further investigated by prospective randomized controlled clinical trials.

### Limitations

This retrospective case-control study has several limitations. First, a retrospective study design is inferior regarding the acquired level of evidence when compared to prospective study designs. However, the incidence of myocarditis is comparatively low, which is why we chose this study design to test our hypotheses. Second, the number of patients enrolled in this study was relatively low (*n* = 114), especially after propensity score matching was conducted (*n* = 54). The number of enrolled patients also explains the scarce follow-up data on LV systolic function (*n* = 29, 25.4% of the enrolled patients), which led to a tighter EF distribution than with index examination. The low number of patients has to be taken into account when the findings of our study are interpreted. Furthermore, our study comprises data from a single study center only. Hence, a large prospective multicenter randomized controlled trial is further warranted to confirm the findings of our study.

## Figures and Tables

**Table 1:** Baseline characteristics, laboratory data and data from ECG and TTE of patients enrolled in the study.

	NSAID (*n* = 39)		No NSAID (*n* = 75)		
Baseline characteristics	median	IQR	median	IQR	*p*-value
Age (years)	29	21–38	37	27–49	0.005
	***%***	***n***	***%***	***n***	***p-value***
Myopericarditis	61.5	24	25.3	19	<0.0001
Male sex	84.6	33	77.3	58	0.463
Diabetes mellitus	0.0	0	1.3	1	0.658
Hyperlipidemia	12.8	5	17.3	13	0.599
Obesity (BMI >30 kg/m^2^)	15.4	6	12.0	9	0.771
Arterial hypertension	10.3	4	17.3	13	0.411
History of smoking	28.2	11	34.7	26	0.533
Coronary artery disease	2.6	1	1.3	1	0.569
Cerebral artery disease	0.0	0	1.3	1	0.658
Peripheral artery disease	0.0	0	0.0	0	NA
Chronic infectious disease	0.0	0	0.0	0	NA
Autoimmune disease	7.1	3	6.9	5	0.621
Active malignancy	2.4	1	1.4	1	0.454
***Laboratory data***	***median***	***IQR***	***median***	***IQR***	***p-value***
Serum creatinine (mg/dL)	0.90	0.78–1.10	0.90	0.80–1.00	0.987
CRP (mg/dL)	7.35	1.45–12.55	1.90	0.60–6.45	0.004
Bilirubin (mg/dL)	0.55	0.43–0.88	0.60	0.40–0.88	0.972
Creatinine kinase (CK) (IU/L)	240	114–528	260	130–451	0.902
CK-MB (%)	9.35	6.98–11.23	10.90	8.30–13.90	0.192
High-sensitivity troponin (hsTnT) (ng/L)	417	195–1043	216	30–584	0.059
Pro brain natriuretic peptide (pBNP) (ng/L)	503	238–1220	278	122–780	0.164
Hemoglobin (mg/dL)	14.60	13.20–15.75	14.65	13.63–15.68	0.964
Leukocyte count (G/L)	9.85	7.62–13.11	7.87	6.49–11.23	0.048
Thrombocyte count (G/L)	210	198–252	219	172–261	0.580
***Initial ECG and TTE***	***%***	***n***	***%***	***n***	***p-value***
ECG changes	76.3	29	68.9	51	0.510
ST-seg. elevation	58.6	17	54.0	27	0.815
ST-seg. depression	24.1	7	24.0	12	0.989
	median	IQR	median	IQR	*p*-value
LV end-diastolic diameter (mm)	48	45–50	48	45–51	0.573
Interv. septum thickness (mm)	11	9–12	11	10–13	0.583
Ejection fraction (%)	56	50–60	55	50–60	0.378

Abbreviations: BMI = body mass index, CRP = C-reactive protein, CK-MB = creatinine kinase muscle-brain type, ECG = electrocardiogram, ST-seg. = ST segment, LV = left ventricular, NSAID = non-steroidal anti-inflammatory drug.

**Table 2:** Data on treatment administered for myocarditis/myopericarditis according to the discharge letter.

	NSAID (*n* = 39)		No NSAID (*n* = 75)		
Treatment for myocarditis/myopericarditis	%	*n*	%	*n*	*p*-value
NSAID	100	39	NA	NA	NA
Acetylsalicylic acid	51.3	20	NA	NA	NA
Ibuprofen	46.2	18	NA	NA	NA
Diclofenac	2.6	1	NA	NA	NA
Colchicine	20.5	8	1.3	1	0.001
Steroid	0.0	0	2.7	2	0.546
Beta blocker	12.8	5	25.3	19	0.150
ACEI/ARB	10.3	4	16.0	12	0.572
MCRA	2.6	1	4.0	3	0.693
	***mean***	***SD***	***mean***	***SD***	***p-value***
Duration of NSAID, weeks	2.9	2.2	NA	NA	NA
Dosage of acetylsalicylic acid per day, mg	1260	395	NA	NA	NA
Dosage of ibuprofen per day, mg	1248	391	NA	NA	NA
Dosage of diclofenac per day, mg	100	NA	NA	NA	NA

Abbreviations: NSAID = non-steroidal anti-inflammatory drug, ACEI = angiotensin-converting enzyme inhibitors, ARB = angiotensin receptor blockers, MRCA = mineralocorticoid receptor antagonists, NA = not applicable.

**Table 3:** Data on the primary and secondary study endpoints in patients of both groups prior to propensity score matching.

	NSAID (*n* = 39)		No NSAID (*n* = 75)		
Primary and secondary study endpoints	%	*n*	%	*n*	*p*-value
12-month mortality	0.0	0	0.0	0	NA
24-month mortality	5.1	2	0.0	0	0.115
Arrhythmias, total	12.8	5	12.0	9	0.899
In-hospital complications	12.8	5	8.0	6	0.507
Admission to IMC/ICU	53.8	21	46.7	35	0.555
	***median***	***IQR***	***median***	***IQR***	***p-value***
EF at follow-up (%)	55	54–60	55	50–56	0.228
FC of EF (ratio)	1.03	0.92–1.21	1.00	0.88–1.14	0.898
Δ EF (%)	1.25	−4.13–8.75	0.00	−7.00–7.75	0.831

Abbreviations: NSAID = non-steroidal anti-inflammatory drug, IMC = intermediate care unit, ICU = intensive care unit, EF = ejection fraction, FC = fold change, NA = not applicable.

## Data Availability

The data underlying this article will be shared on reasonable request to the corresponding author.

## Supplementary Materials

The following supporting information can be downloaded at: https://www.mdpi.com/article/10.3390/jcdd9020032/s1, Figure S1: Distribution of propensity scores (a) before (*n* = 114) and (b) after propensity score matching (*n* = 54); Figure S2: Standardized mean differences of included covariates before and after propensity score matching. Figure S3: Kaplan-Meier plots of all-cause mortality within 24 months of follow-up in patients treated with NSAID segregated by quantiles of C-reactive protein (CRP). Q0 = <5% (<0.10 mg/dL), Q1 = 5–25% (0.10–1.44 mg/dL), Q2 = 25–50% (1.45–7.34 mg/dL), Q3 = 50–75% (7.35–12.54 mg/dL), Q4 = 75–95% (12.55–25.11 mg/dL), Q5 = >95% (>25.11 mg/dL).
