# Supplementary material for: Treatment with Non-Steroidal Anti-Inflammatory Drugs (NSAIDs) Does Not Affect Outcome in Patients with Acute Myocarditis or Myopericarditis
Source: J Cardiovasc Dev Dis. 2022 Jan 19;9(2):32. doi: 10.3390/jcdd9020032 (PMC8880264; doi:10.3390/jcdd9020032)
Supplement: Supplementary file 1 [file jcdd-09-00032-s001.zip › jcdd-1554572-supplementary.pdf]

Treatment with non-steroidal anti-inflammatory drugs (NSAID) does not affect outcome in patients with acute myocarditis or myopericarditis.

- *Supplementary materials* –

Moritz Mirna[1], Lukas Schmutzler[1], Albert Topf[1], Elke Boxhammer[1], Brigitte Sipos[1], Uta C. Hoppe[1], Michael Lichtenauer[1]

[1] Department of Internal Medicine II, Division of Cardiology, Paracelsus Medical University of Salzburg, Austria

Correspondence:

Moritz Mirna, MD, PhD, FESC

Department of Internal Medicine II
Division of Cardiology
Universitätsklinikum der Paracelsus Medizinischen Universität
Müllner Hauptstraße 48
5020 Salzburg
Austria

email: m.mirna@salk.at

Telephone: +43 (0) 57255 - 58340

ORCID iD: https://orcid.org/0000-0001-5679-4872

Supplementary Figure S1

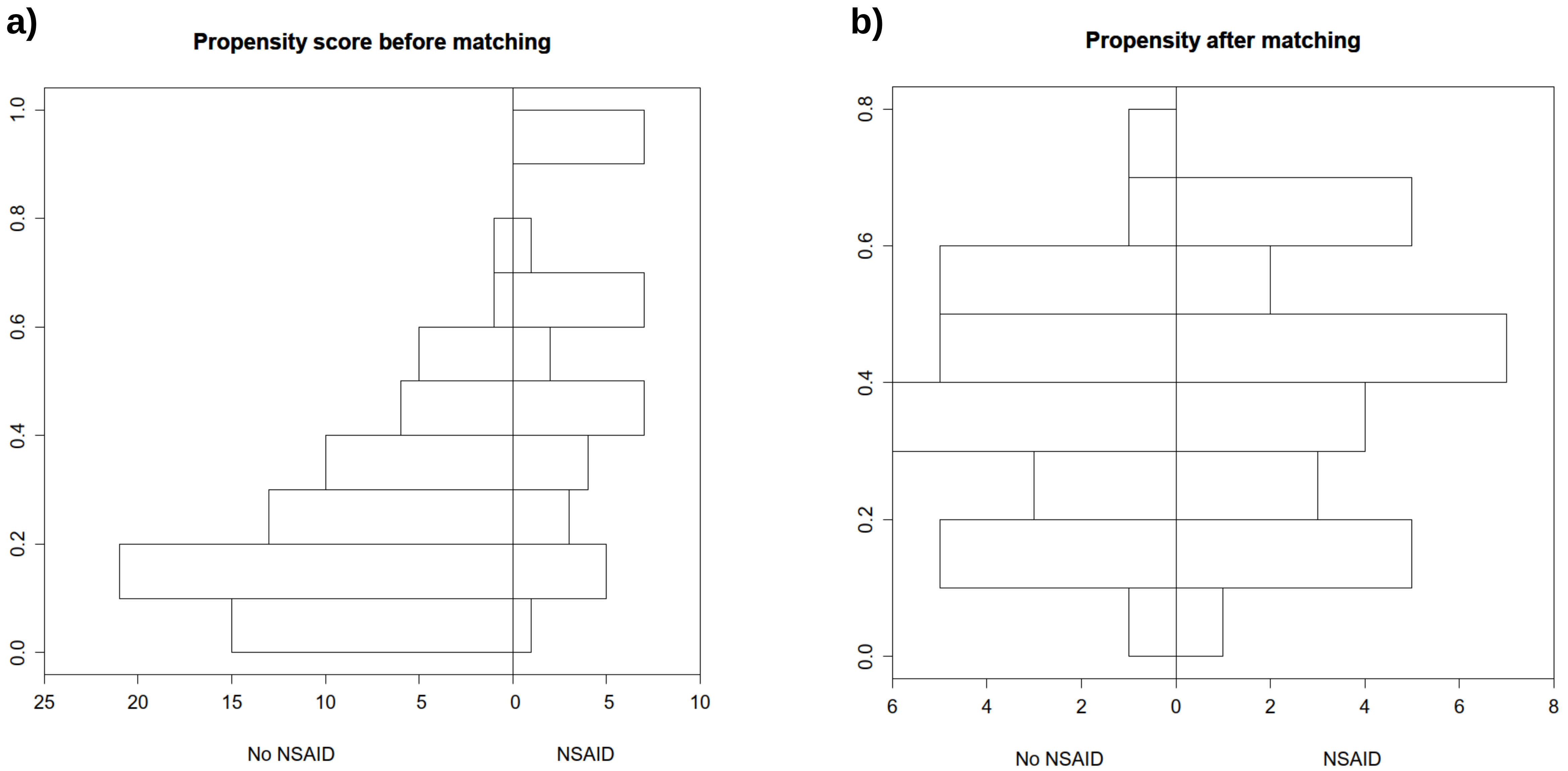

Suppl. Figure S1: Distribution of propensity scores **a)** before (n=114) and **b)** after propensity score matching (n= 54). *Abbreviations: NSAID= non-steroidal anti-inflammatory drug.*

Supplementary Figure S2

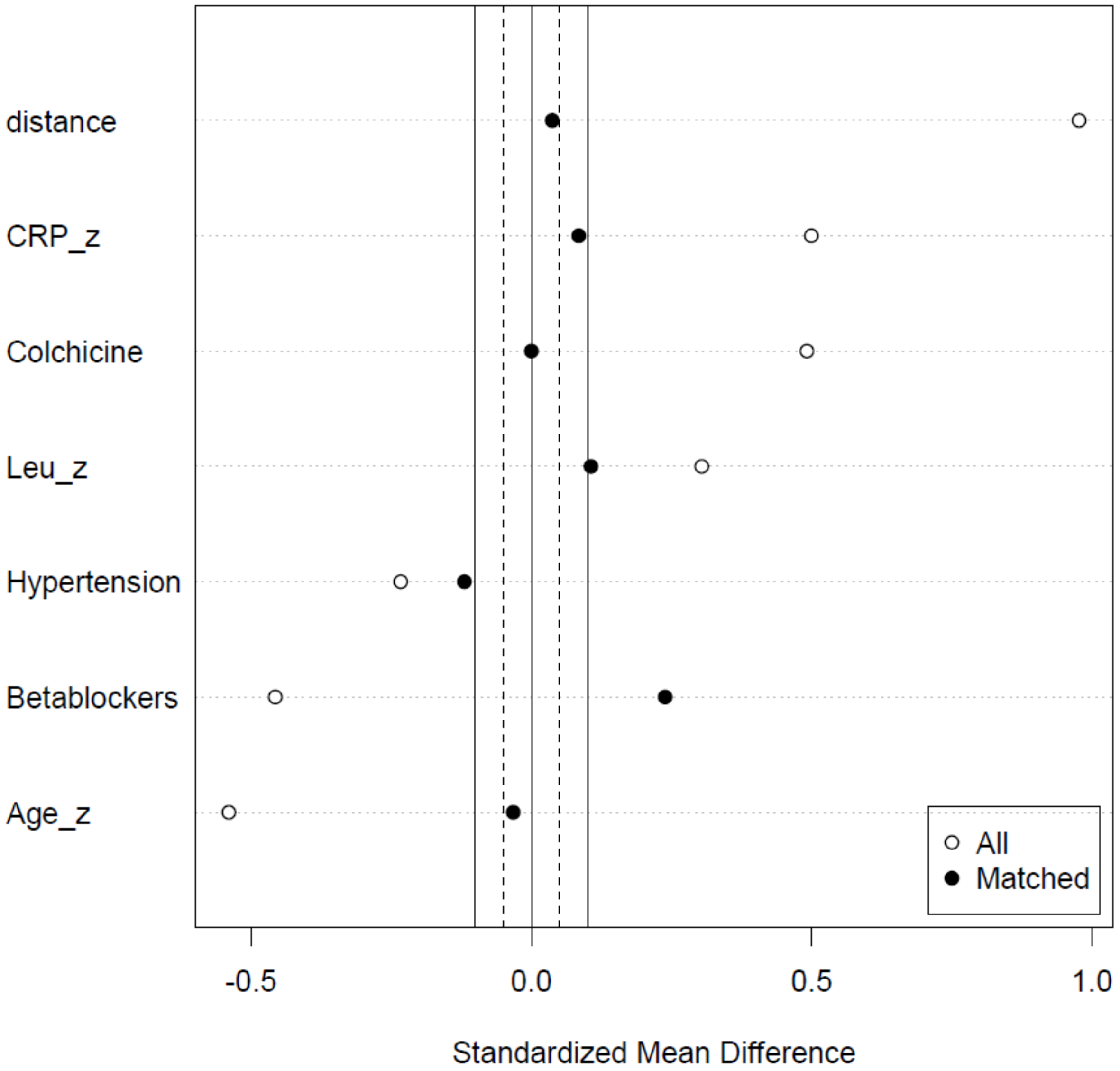

Suppl. Figure S2: Standardized mean differences of included covariates before and after propensity score matching. *Abbreviations: CRP_z= calculated z-score of C-reactive protein, Leu_z= calculated z-score of leukocyte count.*

Supplementary Figure S3

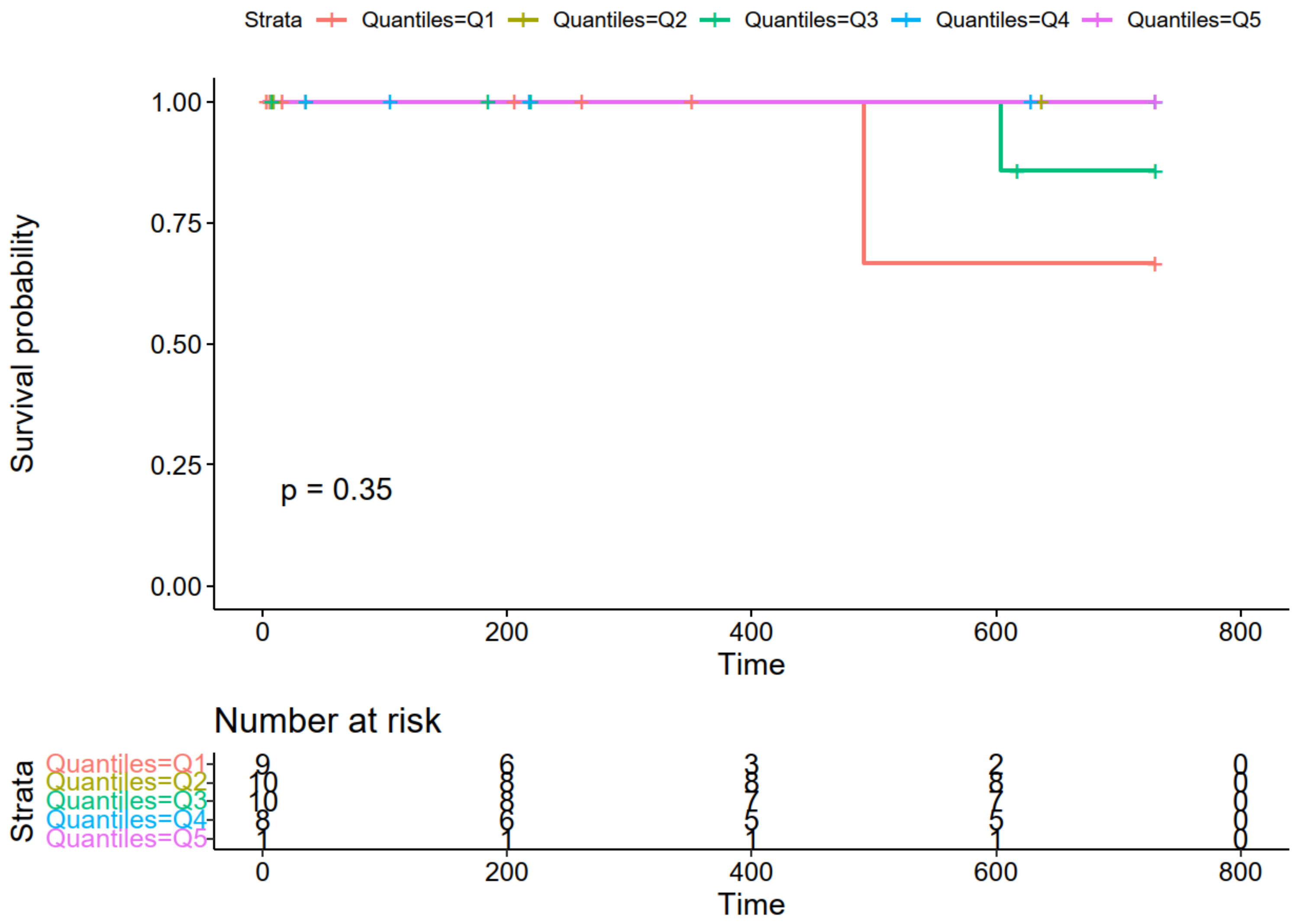

Suppl. Figure S3: Kaplan-Meier plots of all-cause mortality within 24 months of follow-up in patients treated with NSAID segregated by quantiles of C-reactive protein (CRP). Q0= <5% (<0.10 mg/dl), Q1= 5-25% (0.10-1.44 mg/dl), Q2= 25-50% (1.45-7.34 mg/dl), Q3= 50-75% (7.35-12.54 mg/dl), Q4= 75-95% (12.55-25.11 mg/dl), Q5= >95% (>25.11 mg/dl)
